# Supplementary material for: Targeting microbial bile salt hydrolase reprograms bile acid metabolism and ameliorates metabolic dysfunction–associated steatohepatitis in mice
Source: Mol Metab. 2026 Aug 18;112:102430. doi: 10.1016/j.molmet.2026.102430 (PMC13571960; doi:10.1016/j.molmet.2026.102430)
Supplement: Supplementary file 1 [file mmc1.docx]

**Supplemental Table1. Metal-conjugated antibody panel used for CyTOF analysis**

| **Metal Isotope** | **Marker** | **Staining Type** | **Clone** | **Vendor** | **catalog number** |
| --- | --- | --- | --- | --- | --- |
| 145Nd | CD69 | surface stain | H1.2F3 | Standard biotools | 3145005B |
| 160Gd | CD62L/L-selectin | surface stain | MEL-14 | Standard biotools | 3160008B |
| 151Eu | CD25/IL-2R | surface stain | 3C7 | Standard biotools | 3151007B |
| 149Sm | CD19 | surface stain | 6D5 | Standard biotools | 3149002B |
| 152Sm | CD3 epsilon | surface stain | 145-2C11 | Standard biotools | 3152004B |
| 154Sm | TER-119/Glycophorin A | surface stain | TER-119 | Standard biotools | 3154005C |
| 176Yb | CD45R/B220 | surface stain | RA3-6B2 | Standard biotools | 3176002B |
| 170Er | CD161/NK1.1 | surface stain | PK136 | Standard biotools | 3170002B |
| 142Nd | CD11c | surface stain | N418 | Standard biotools | 3142003B |
| 169Tm | TCR beta | surface stain | H57-597 | Standard biotools | 3169002B |
| 148Nd | CD11b/Mac-1 | surface stain | M1/70 | Standard biotools | 3148003B |
| 168Er | CD8a | surface stain | 53-6.7 | Standard biotools | 3168003B |
| 172Yb | CD4 | surface stain | RM4-5 | Standard biotools | 3172003B |
| 147Sm | CD45 | surface stain | 30-F11 | Standard biotools | 3147003B |
| 141Pr | Ly-6G/C/Gr-1 | surface stain | RB6-8C5 | Standard biotools | 3141005B |
| 171Yb | CD44 | surface stain | IM7 | Standard biotools | 3171003B |
| 159Tb | CD279/PD-1 | surface stain | 29F.1A12 | Standard biotools | 3159024B |
| 155Gd | CD186(CXCR6) | surface stain | SA051D1 | Standard biotools | Custom-conjugated |
| 198Pt | Granzyme B | cytokine stain | GB11 | Standard biotools | 3198002B |
| 165Ho | IFN gamma | cytokine stain | XMG1.2 | Standard biotools | 3165003B |
| 144Nd | IL-2 | cytokine stain | JES6-5H4 | Standard biotools | 3144002B |
| 166Er | IL-4 | cytokine stain | 11B11 | Standard biotools | 3166003B |
| 174Yb | IL-17A | cytokine stain | TC11-18H10.1 | Standard biotools | 3174002B |
| 162Dy | TNF alpha | cytokine stain | MP6-XT22 | Standard biotools | 3162002B |
| 167Er | IL-6 | cytokine stain | MP5-20F3 | Standard biotools | 3167003B |
| 158Gd | CD9 | cytokine stain | KMC8 | Standard biotools | 3158009B |
| 146Nd | F4/80 | cytokine stain | BM8 | Standard biotools | 3146008B |
| 153Eu | CD335/NKp46 | cytokine stain | 29A1.4 | Standard biotools | 3153006B |
